# Development and Evaluation of Active Case Detection Methods to Support Visceral Leishmaniasis Elimination in India

**DOI:** 10.3389/fcimb.2021.648903

**Published:** 2021-03-24

**Authors:** Pushkar Dubey, Aritra Das, Khushbu Priyamvada, Joy Bindroo, Tanmay Mahapatra, Prabhas Kumar Mishra, Ankur Kumar, Ana O. Franco, Basab Rooj, Bikas Sinha, Sreya Pradhan, Indranath Banerjee, Manash Kumar, Nasreen Bano, Chandan Kumar, Chandan Prasad, Parna Chakraborty, Rakesh Kumar, Niraj Kumar, Aditya Kumar, Abhishek Kumar Singh, Kumar Kundan, Sunil Babu, Hemant Shah, Morchan Karthick, Nupur Roy, Naresh Kumar Gill, Shweta Dwivedi, Indrajit Chaudhuri, Allen W. Hightower, Lloyd A C. Chapman, Chandramani Singh, Madan Prasad Sharma, Neeraj Dhingra, Caryn Bern, Sridhar Srikantiah

**Affiliations:** ^1^ Bihar Technical Support Program, CARE-India Solutions for Sustainable Development, Patna, India; ^2^ Institute for Global Health Sciences, Department of Epidemiology and Biostatistics, University of California San Francisco, San Francisco, CA, United States; ^3^ National Vector Borne Disease Control Programme, Ministry of Health and Family Welfare, Government of India, Delhi, India; ^4^ Independent Consultant, Bangkok, Thailand; ^5^ Department of Medicine, University of California, San Francisco, San Francisco, CA, United States; ^6^ Centre for Mathematical Modelling of Infectious Disease, London School of Hygiene and Tropical Medicine, London, United Kingdom; ^7^ Department of Community and Family Medicine, All India Institute of Medical Sciences, Patna, India; ^8^ Department of Health, Government of Bihar, Patna, India

**Keywords:** active case detection, visceral leishmaniasis, visceral leishmaniasis elimination, evaluation of active case detection, India, surveillance

## Abstract

As India moves toward the elimination of visceral leishmaniasis (VL) as a public health problem, comprehensive timely case detection has become increasingly important, in order to reduce the period of infectivity and control outbreaks. During the 2000s, localized research studies suggested that a large percentage of VL cases were never reported in government data. However, assessments conducted from 2013 to 2015 indicated that 85% or more of confirmed cases were eventually captured and reported in surveillance data, albeit with significant delays before diagnosis. Based on methods developed during these assessments, the CARE India team evolved new strategies for active case detection (ACD), applicable at large scale while being sufficiently effective in reducing time to diagnosis. Active case searches are triggered by the report of a confirmed VL case, and comprise two major search mechanisms: 1) case identification based on the index case’s knowledge of other known VL cases and searches in nearby houses (snowballing); and 2) sustained contact over time with a range of private providers, both formal and informal. Simultaneously, house-to-house searches were conducted in 142 villages of 47 blocks during this period. We analyzed data from 5030 VL patients reported in Bihar from January 2018 through July 2019. Of these 3033 were detected passively and 1997 *via* ACD (15 (0.8%) *via* house-to-house and 1982 (99.2%) by light touch ACD methods). We constructed multinomial logistic regression models comparing time intervals to diagnosis (30-59, 60-89 and ≥90 days with <30 days as the referent). ACD and younger age were associated with shorter time to diagnosis, while male sex and HIV infection were associated with longer illness durations. The advantage of ACD over PCD was more marked for longer illness durations: the adjusted odds ratios for having illness durations of 30-59, 60-89 and >=90 days compared to the referent of <30 days for ACD vs PCD were 0.88, 0.56 and 0.42 respectively. These ACD strategies not only reduce time to diagnosis, and thus risk of transmission, but also ensure that there is a double check on the proportion of cases actually getting captured. Such a process can supplement passive case detection efforts that must go on, possibly perpetually, even after elimination as a public health problem is achieved.

## Introduction

Complete and timely case detection has become increasingly crucial as India moves toward elimination of visceral leishmaniasis (VL) as a public health problem, defined as a target incidence of less than 1 case per 10,000 population per year at the sub-district level (Rijal et al., 2019). Households with high VL attack rates disproportionately come from the most disadvantaged segments of Indian society (Boelaert et al., 2009). People living in high-risk areas often have limited knowledge about the disease (Hirve et al., 2010; Khatun et al., 2014; Govil et al., 2018). Lack of knowledge impedes treatment-seeking behavior, while poverty and societal disadvantage may lead patients to consult local unqualified practitioners before applying to public health clinics, thereby delaying appropriate treatment (Hasker et al., 2010; Khatun et al., 2014; Boettcher et al., 2015). The longer the delay in receiving effective treatment, the higher the likelihood of onward community transmission (Medley et al., 2015).

When CARE began providing support to the VL program in eight of the affected districts of Bihar in 2013, VL cases were found primarily through passive case detection (PCD) (Huda et al., 2012). In the years leading up to that point, research studies covering localized populations claimed that reported VL incidence represented 8-fold and 4-fold underestimates for the years 2003 and 2006, respectively (Singh et al., 2006; Singh et al., 2010). These findings posed a serious potential problem to the elimination program, since IRS, the main strategy for prevention and control, was mandated to cover all villages affected by VL cases in the previous three years; accurate targeting required comprehensive VL case data.

To more accurately estimate the extent of under-reporting, CARE conducted broad evaluations in 8 endemic districts of Bihar in 2013, followed by similar evaluations in all 33 endemic districts of Bihar and the 4 endemic districts of Jharkhand in 2015. This article presents the methodology and results of these evaluations, and the way they were used to develop new strategies for active case detection (ACD) of VL applicable at large scale. We then used VL surveillance data generated since the implementation of these ACD activities to evaluate the effectiveness of the strategy. In the effectiveness analysis, we focus on time to diagnosis rather than discovery of additional cases, because the 2013-2015 assessments indicated that the vast majority of cases were eventually captured by the public health system.

## Methods

### Study Area

Bihar and Jharkhand are among the poorest states in India. According to the census of India 2011, Bihar had a population of 104 million, population density of 1106 per square km, 61.8% literacy, 88.7% rural population, and 1.3% and 15.9% of the population belonging to scheduled tribes (STs) and scheduled castes (SCs) respectively. Of Bihar’s 38 districts, 33 are considered endemic for VL. Bihar accounted for approximately 72% of total VL cases in 2017.

Jharkhand came into existence as the 28th state of India in 2000 when its 24 districts were separated from Bihar. In the census of India 2011, the state had a population of 33 million, population density of 414 per square km, 66.4% literacy, 75.9% rural population, and 26.2% and 12.1% of the population belonging to scheduled tribes (STs) and scheduled castes (SCs) respectively. The four endemic districts of Jharkhand accounted for approximately 24% of the total VL burden in 2017.

### Methods for the 2013 and 2015 Assessments

The assessment methodology used in 2013 and 2015 built upon the lessons from a previous study commissioned by the World Bank and the National Vector Borne Disease Control Program (NVBDCP), which sought to develop a practical alternative to house-to-house surveys for accurately estimating annual VL incidence at the block level. The sample sizes for attaining a reasonably precise estimate of the very low, clustered incidence of VL in a house-to-house survey verged on being equal to the universe of villages in a block. Such surveys were too expensive and virtually impossible to carry out in multiple blocks with fidelity. The proposed alternative was the snowballing technique, previously used to estimate incidence of even rarer events like maternal mortality (Singh et al., 2007). This method was expected to work for VL, since the existence of undetected VL cases, either alive or dead, would be widely known to others in the same village as someone who had suffered a prolonged illness. Once such a ‘suspect’ was identified, the concerned family could be approached for details and evidence of diagnosis and/or treatment.

The results of the World Bank/NVDBCP study that compared snowballing to house-to-house surveys were available in 2013, although not published until 2016 (Siddiqui et al., 2016), and suggested that snowballing could detect most VL cases not reported to the program, particularly if augmented by other commonsense approaches. For instance, in the initial study, field investigators were blinded to known VL cases to avoid bias, but field experience suggested that VL patients often know about other VL cases. Thus, index VL cases could act as useful starting points, along with other potentially knowledgeable local informants, such as resident village health workers such as Accredited Social Health Activists (ASHA) and Anganwadi Workers (AWW), informal and formal private providers serving the village, local leaders and shopkeepers. These lessons informed the methodology adopted by CARE in the assessments of VL incidence in 2013 and 2015.

#### The 2013 Assessment

**Figure 1:**
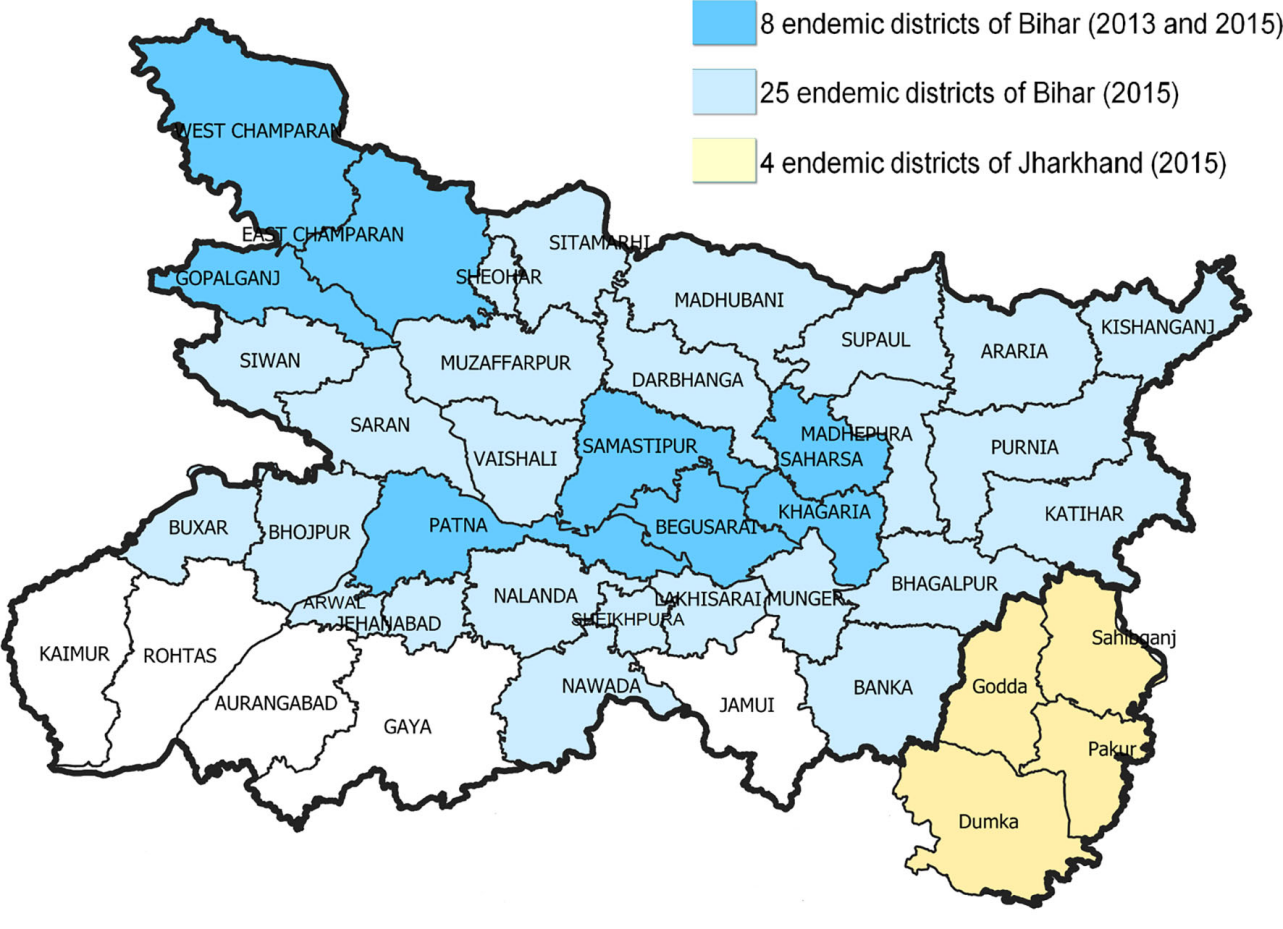
Districts included in the 2013 and 2015 assessments of visceral leishmaniasis under-reporting. The 33 visceral leishmaniasis-endemic districts of Bihar are shown in blue, while the 4 endemic districts of Jharkhand are in yellow. Districts in darker blue were included in both assessments.

This assessment aimed to estimate VL incidence and under-reporting from January 2012 to June 2013, in all 137 blocks of 8 districts of Bihar (**Figure 1**). All available VL case line lists were obtained from the state program office and multiple government facilities across the affected districts, and reconciled to eliminate duplicates. These cleaned lists constituted the index cases. CARE field teams, trained for the purpose, then attempted to trace and interview each index case at home. Those found were queried about patients with recognized VL or PKDL, persons known to have had prolonged illness, and those who had died from known VL or other prolonged illnesses during the reference period. Field investigators then walked to each distinct hamlet in the village and asked potential local informants about possible VL cases, alive or dead, using a standardized set of probe questions. Houses in the immediate neighborhood of the index case were visited to ask similar questions, after an introduction to explain that there were known cases of kala-azar in the village and the study was aimed at uncovering additional cases. These questions were asked regarding any such individuals known to the interviewees, whether in the same village or elsewhere. All suspects found in this manner were listed along with contact details in a common roster format. If suspected cases belonged to the same village, an attempt was made to contact them immediately and interview them for evidence of VL diagnosis and treatment during the reference period. Each was also asked whether they knew about other VL cases, and these names and details were added to the roster. This continued until no further leads remained to be followed up in that village.

Lists of suspects belonging to other villages were added to a district level compilation, and if necessary, to a central compilation. Lists sorted by address were sent to the field teams in charge of the corresponding geographies to be similarly followed up. While this approach covered all villages where traceable index cases resided, these constituted a minority of all villages of the 137 blocks. Leads obtained from these villages also led to other villages not previously known to be affected. In addition, lists were made of all private labs, pharmacies, doctors and hospitals in each block and district headquarters town, and to the extent possible, a team member visited each of them and requested information regarding any VL case known to them in the reference period. Rural health practitioners (RHPs), including those without formal qualifications, were included if they had a sizeable clientele. Names of possible cases obtained in this manner were added to the list of suspects. All listed suspects from all sources were tracked until found or abandoned as untraceable after a reasonable effort. The entire exercise took over three months to complete.

For the purposes of analysis, a confirmed VL case was defined as a case that was found in any of the line lists maintained by a public facility, having a date of diagnosis within the reference period and traced to his/her residence in one of the 137 blocks; or, in the case of additional cases found, as a case having documentary evidence of diagnosis by any laboratory method, or a prescription for a drug known to be used in the treatment of kala azar, with a date of testing or treatment within the reference period and residence in any of the 137 blocks. The number of additional cases found was expressed as a percentage of the total cases found. Upper and lower error limits of these numbers were computed based on the possible errors in case identification, and under-reporting was expressed as a percentage range.

#### The 2015 Assessment

By 2015, CARE had moved forward to support the kala-azar elimination program in all 33 affected districts in Bihar and the four affected districts of Jharkhand. An assessment was conducted covering all 37 districts, to find and trace unreported cases and to identify villages left out of interventions. Field experiences from the 2013 study were utilized to refine interview tools and operational details in tracking. The questions used for identifying suspects in the field and the case definitions remained the same. Detailed case interviews primarily aimed to understand care-seeking patterns, to inform refinements to case-finding methods and to evolve strategies to reduce time to diagnosis. The reference period for this assessment was July 2013 to December 2014, and under-reporting ranges estimated using the same method as in the 2013 assessment.

### Active Case Detection Methods Implemented in 2017

**Figure 2:**
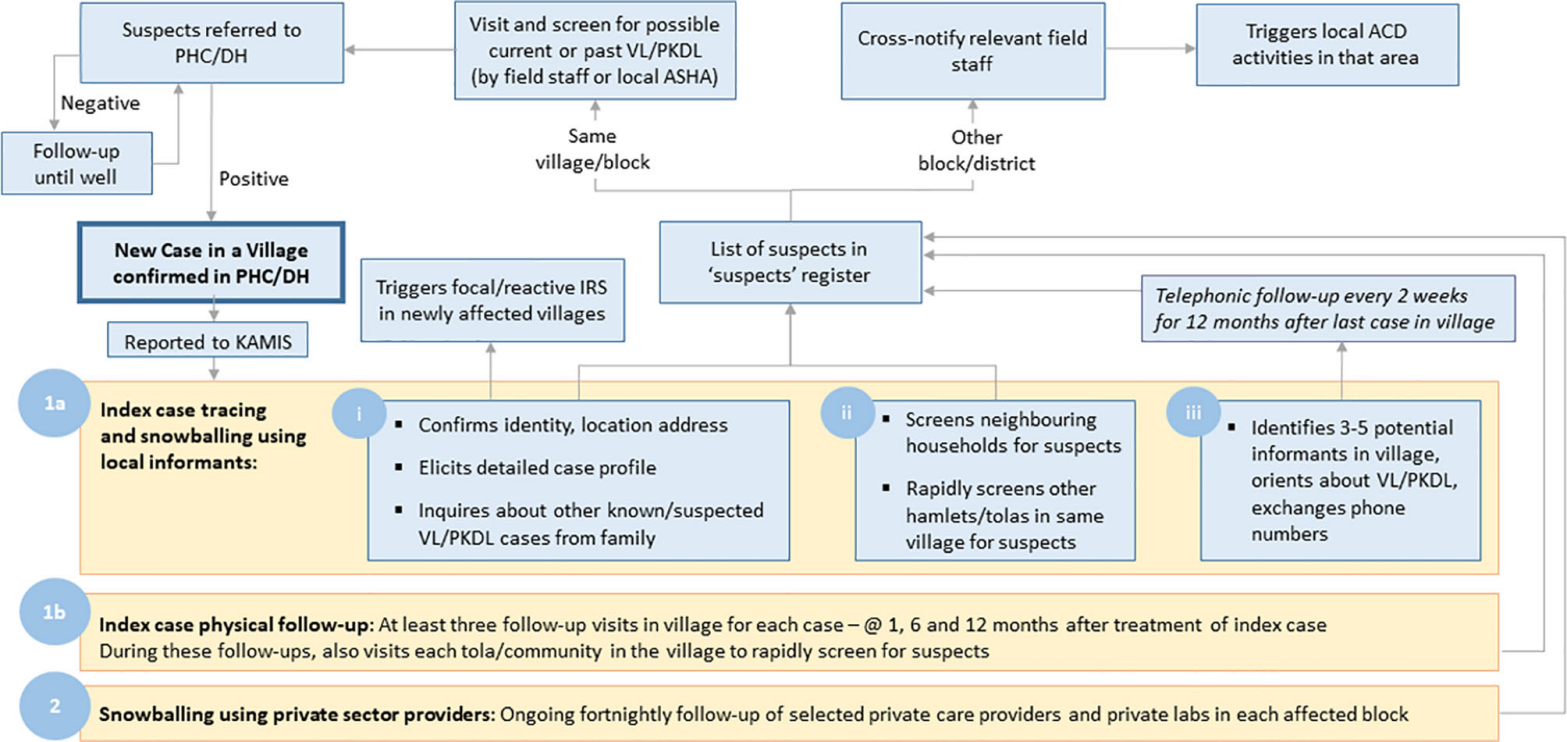
Schematic of the steps involved in active case detection linked to the Kala-azar Management Information System (KAMIS). Active case searches are triggered by the report of a new confirmed visceral leishmaniasis case. Searches employ snowballing based on information from the index case and knowledgeable local informants regarding other nearby case, periodic follow-up of the index case, and repeated contacts over time with local private health care providers.

Since 2016, Kala-Azar Block Coordinators (KBCs) deployed by CARE have been responsible for facilitating appropriate VL diagnosis and treatment at the nearest public health facility (PHC), and for coordinating ACD efforts in their respective blocks (subdistricts). The ACD method evaluated here represents a refinement of those developed in the 2013 and 2015 VL assessments. Active case searches are triggered by the report of a confirmed VL case, and follow two major search mechanisms: 1) case identification based on the index case’s knowledge of other known VL cases and searches in nearby houses with the help of knowledgeable local informants (snowballing); and 2) sustained contact over time with a range of private providers, both formal and informal (**Figure 2**). The methods were modified to enable application at high frequency while remaining scalable in program settings.

As currently conducted, the KBC visits the village within seven days of a new VL case confirmation, interviews the patient and identifies key informants. Key informants in the village include ASHAs, AWWs, RHPs, other willing community members, and members of Community Based Organizations. In addition, selected Private Practitioners (PP), chemists and diagnostic labs in the block or district headquarters town are identified. All informants are oriented at the outset about symptoms of VL and PKDL, using simple communications material and requested to maintain watch for such patients. The KBC maintains fortnightly contact with key informants *via* mobile phone and/or personal visits for 12 months after the last case in a targeted village, and perpetually with identified health care providers in towns. Since January 2017, the described ACD activities have been conducted across all affected blocks, targeting villages that had reported a case in the previous 12 months. In addition to the light-touch methods described here, house-to-house searches were conducted during the same time period by ASHAs trained for the purpose, using standardized data collection and reporting protocols.

### Methods for 2018-2019 ACD Effectiveness Evaluation

#### Data Sources

KBCs maintain a suspect VL case registry book listing contact details, date on which the patient was first reported as a suspect, and the referral source; this registry was the data source for the ‘suspect’ date. The diagnosis date was extracted from Kala-azar Management Information System (KAMIS), the official reporting mechanism for health care facilities that perform confirmatory diagnostic testing for VL. As part of routine surveillance activities, KBCs interview all VL patients using the Case Details Form (CDF) as soon as they are confirmed by the PHC and reported to KAMIS. The CDF is a structured validated questionnaire that includes demographic information, caste, occupation, house type, migration for work, and detailed histories of symptoms and treatment seeking. The KAMIS ID, a unique patient identifier assigned at the time of diagnosis and verified through internal data checks, was used to merge the three data sets (suspect case register, KAMIS, CDF). Inconsistencies, duplication, and missing values in the data were reconciled by repeated field validations. Data quality was assured through spot checks and back checks.

#### ACD Case Definitions and Data Analysis

A patient whose reporting date in the suspect register was on or before the date of diagnosis in KAMIS was classified as having been identified through ACD. Patients not listed in the suspect register and those for whom the date of diagnosis preceded the date listed in the suspect case register were classified as PCD. VL patients detected by ACD in the house-to-house surveys conducted during the study period were not distinguishable in the CDF data, but the number of such cases was available by month of report.

Analyses were performed using days from fever onset to diagnosis classified in four categories (<30 days, 30-59 days, 60-89 days and ≥90 days). Variables potentially associated with time from illness onset to diagnosis were tested first in univariable multinomial logistic regression models, and finally combined in a multivariable model. An additional multivariable logistic regression model was constructed for the binary outcome of illness duration ≥180 days vs <180 days to test factors associated with very prolonged illness. Multivariable models were constructed in a forward stepwise manner, including age categories and sex in all models and using p<0.05 as the threshold for maintaining other variables in the model. SAS version 9.4 was used for all statistical procedures.

## Results

### The 2013 Assessment

**Figure 3:**
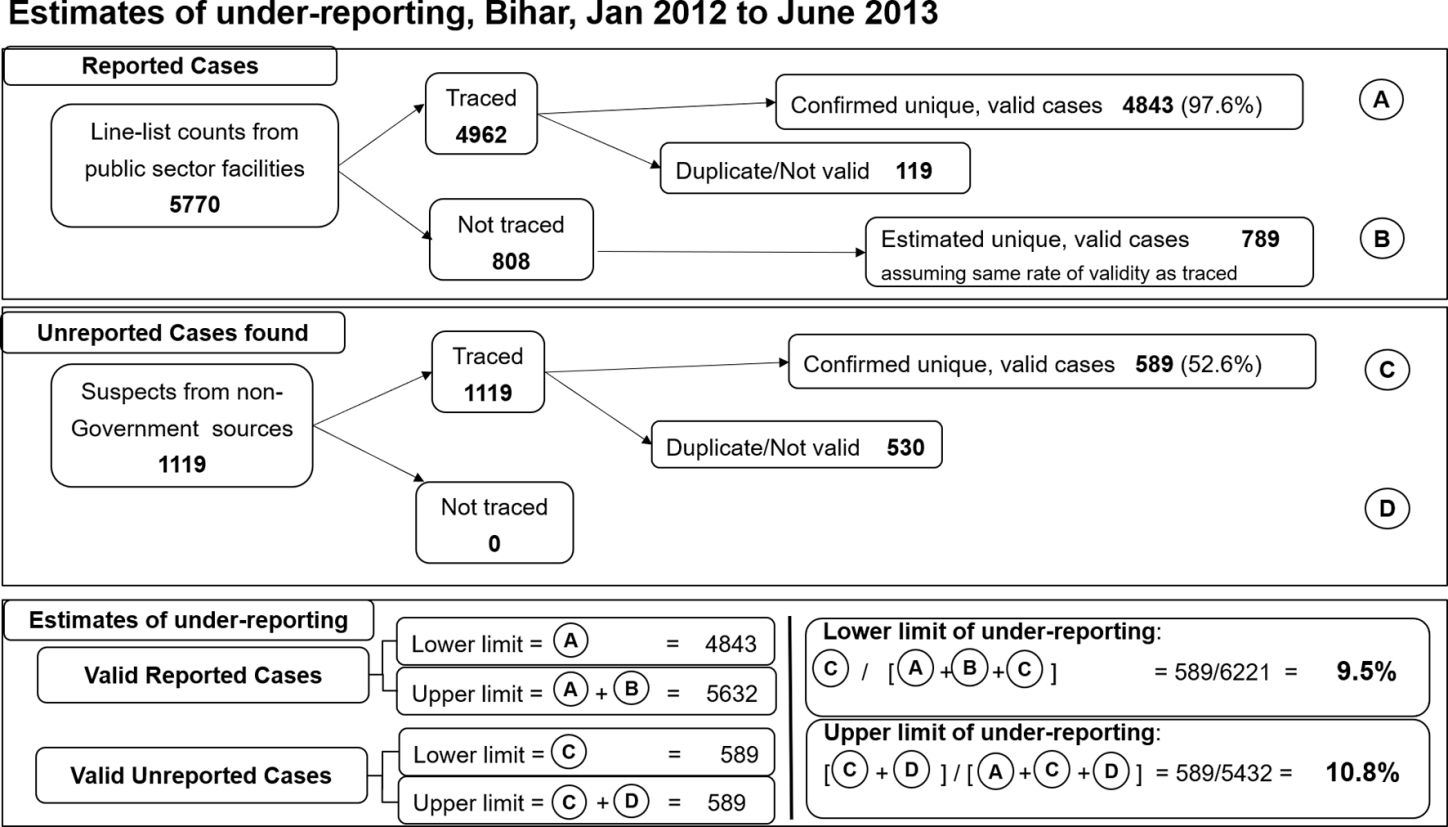
Estimates of visceral leishmaniasis under-reporting, 8 districts of Bihar, January 2012-June 2013. A total of 5770 cases were extracted from the line list of visceral leishmaniasis cases treated at public sector facilities during the study period, of which 4962 were traced and 4843 of those validated **(A)**. Assuming the untraced cases had the same rate of validity as the traced cases yields an additional 734 potentially valid cases **(B)**. From non-government sources, all 1119 suspected cases were traced and 589 validated as unreported cases **(C)**. There were no untraced cases **(D)**. Based on these data, the validated case figure was estimated to range between 4843 and 5632 (A to A+B), while unreported case figure was 589 (C; D=0). Using the extremes of these estimated ranges, underreporting was estimated between 9.5% and 10.8%.

A total of 5770 VL cases were identified in patient lists derived from public health care facilities in the eight districts assessed, with dates of diagnosis in the reference period of Jan 2012 to June 2013 (**Figure 3**). Of these, 4962 cases were successfully traced to their residence. Records of 1119 VL cases were obtained from private facilities, through snowballing and from other local informants. Following removal of duplicate listings, 5432 eligible cases were identified, including 589 cases not previously identified in any data system. The extent of under-reporting of VL cases estimated to fall between 9.5% and 10.8%.

### The 2015 Assessment

**Figure 4:**
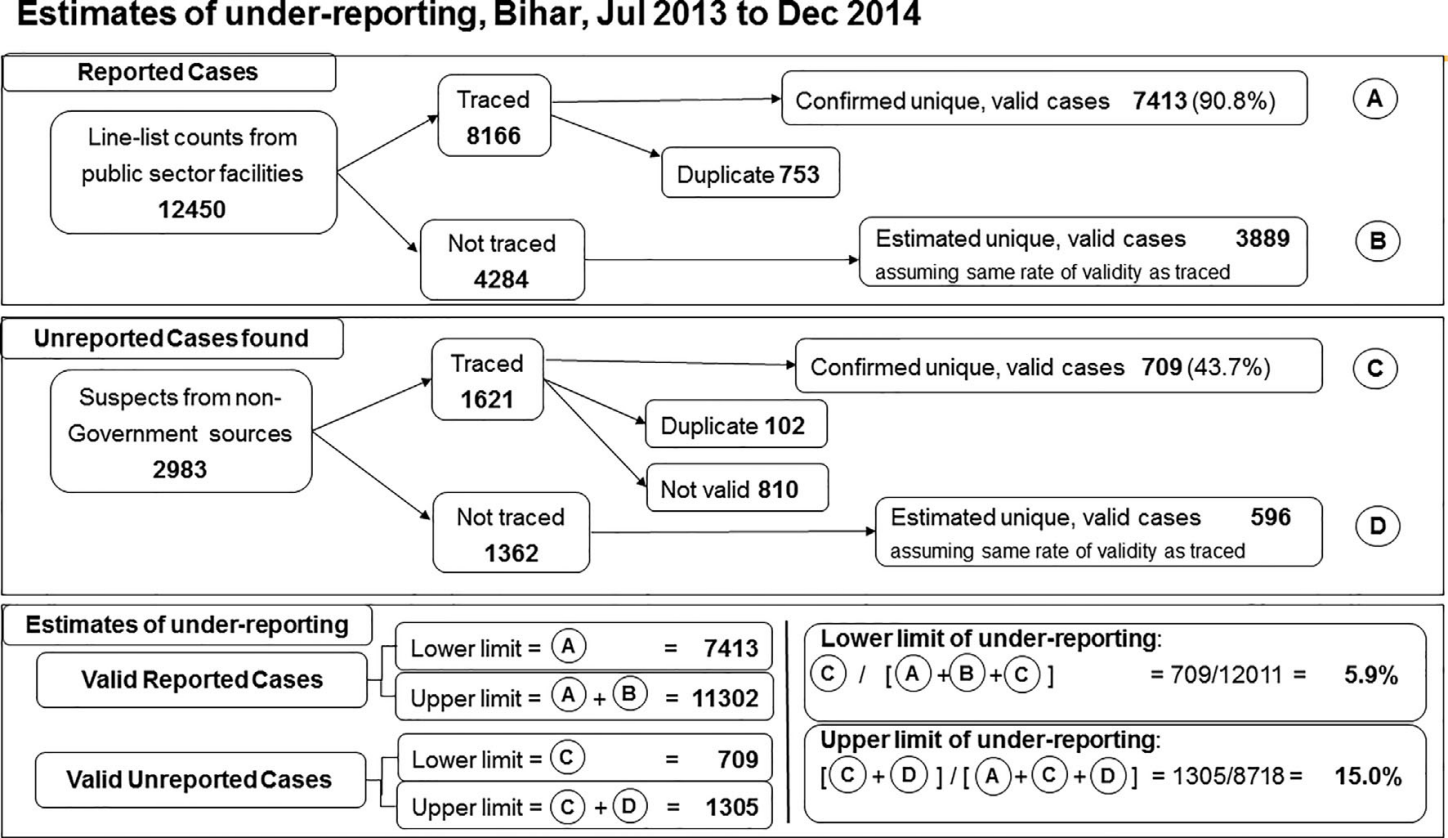
Estimates of visceral leishmaniasis under-reporting, 33 endemic districts of Bihar, July 2013-December 2014. A total of 12,450 cases were extracted from the line list of visceral leishmaniasis cases treated at public sector facilities during the study period, of which 8166 were traced and 7413 of those validated **(A)**. Assuming the untraced cases had the same rate of validity as the traced cases yields an additional 3889 potentially valid cases **(B)**. From non-government sources 2983 suspected cases were identified, of which 1621 were traced and 709 validated as unreported cases **(C)**. Assuming the same rate of validity as for traced suspects yields 596 potentially valid unreported cases **(D)**. Based on these data, the validated case figure was estimated to range between 7413 and 13,013 (A to A+B), while unreported case figure was between 709 and 1305 (C to C+D). Using the extremes of these estimated ranges, underreporting was estimated between 5.9% and 15.0%.

**Figure 5:**
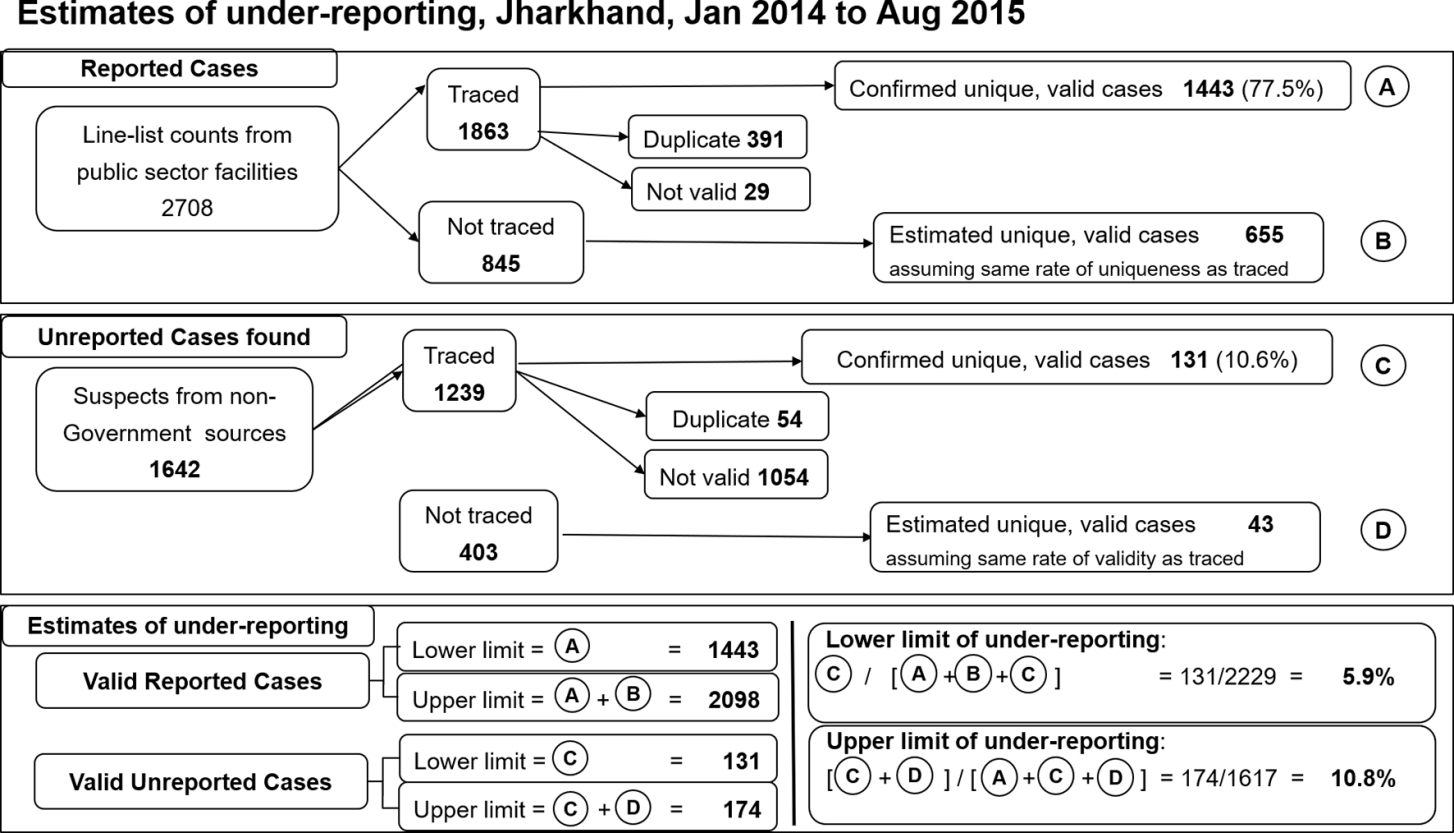
Estimates of visceral leishmaniasis under-reporting, 4 endemic districts of Jharkhand, July 2013-December 2014. A total of 2708 cases were extracted from the line list of visceral leishmaniasis cases treated at public sector facilities during the study period, of which 1863 were traced and 1443 of those validated **(A)**. Assuming the untraced cases had the same rate of validity as the traced cases yields an additional 655 potentially valid cases **(B)**. From non-government sources 1642 suspected cases were identified, of which 1239 were traced and 131 validated as unreported cases **(C)**. Assuming the same rate of validity as for traced suspects yields 43 potentially valid unreported cases **(D)**. Based on these data, the validated case figure was estimated to range between 1443 and 2098 (A to A+B), while unreported case figure was between 131 and 174 (C to C+D). Using the extremes of these estimated ranges, underreporting was estimated between 5.9% and 10.8%.

In Bihar, public sector facility line lists contained 12,450 patients diagnosed within the reference period of July 2013 to December 2014 (**Figure 4**). Of these, 8166 were traced; 753 of these were found to be duplicate listings, yielding 7413 unique valid VL cases (90.8% of those traced). We were unable to trace 4284 of the listed patients; if the duplicate rate was equivalent for these cases, an additional 3889 cases might have occurred. A total of 2983 suspected cases were listed from all other sources of information tapped; 1621 were traced and 1362 were not traceable. Of the 1621 traced cases, 102 were duplicates, 810 did not meet the criteria for a valid VL case, yielding 709 (43.7%) confirmed cases from these sources. If the rate of validation was equivalent for the untraced cases, this would yield an estimated 596 additional cases. Using confirmed but previously unreported cases to calculate the lower limit and confirmed plus estimated unreported cases to calculate the upper limit of under-reporting, this yields between 5.9 and 15% under-reporting during this period of time. The same method yields an estimate of between 5.9 and 10.8% under-reporting for Jharkhand (**Figure 5**).

The inference from these evaluations was that more than 80% of all VL cases were sooner or later getting captured and reported by the elimination program, and that underreporting was much less than that projected by the localized studies performed a decade earlier (Singh et al., 2006; Singh et al., 2010).

### 2018-2019 ACD Effectiveness

We included data from the first 19 months of full ACD implementation in Bihar, comprising VL patients diagnosed between January 1, 2018 and July 31, 2019. A total of 5281 VL patients were recorded in KAMIS during the study period. Case details form (CDF) data were missing for 251 (4.8%) patients. There was no difference in age distribution for included vs excluded patients, but males were slightly more likely than females to have missing data (**Supplemental Table S1**). Those from marginalized castes were slightly less likely to have missing CDF data. Although the numbers of each were small, those with HIV infection or a history of prior VL treatment were significantly more likely to have missing CDF data, possibly because they were more likely to be treated at referral centers rather than within the main network of government primary care facilities. Completeness increased significantly in more recent time periods compared to earlier ones, from 93% in the first half of 2018 to more than 98% in 2019.

**Figure 6:**
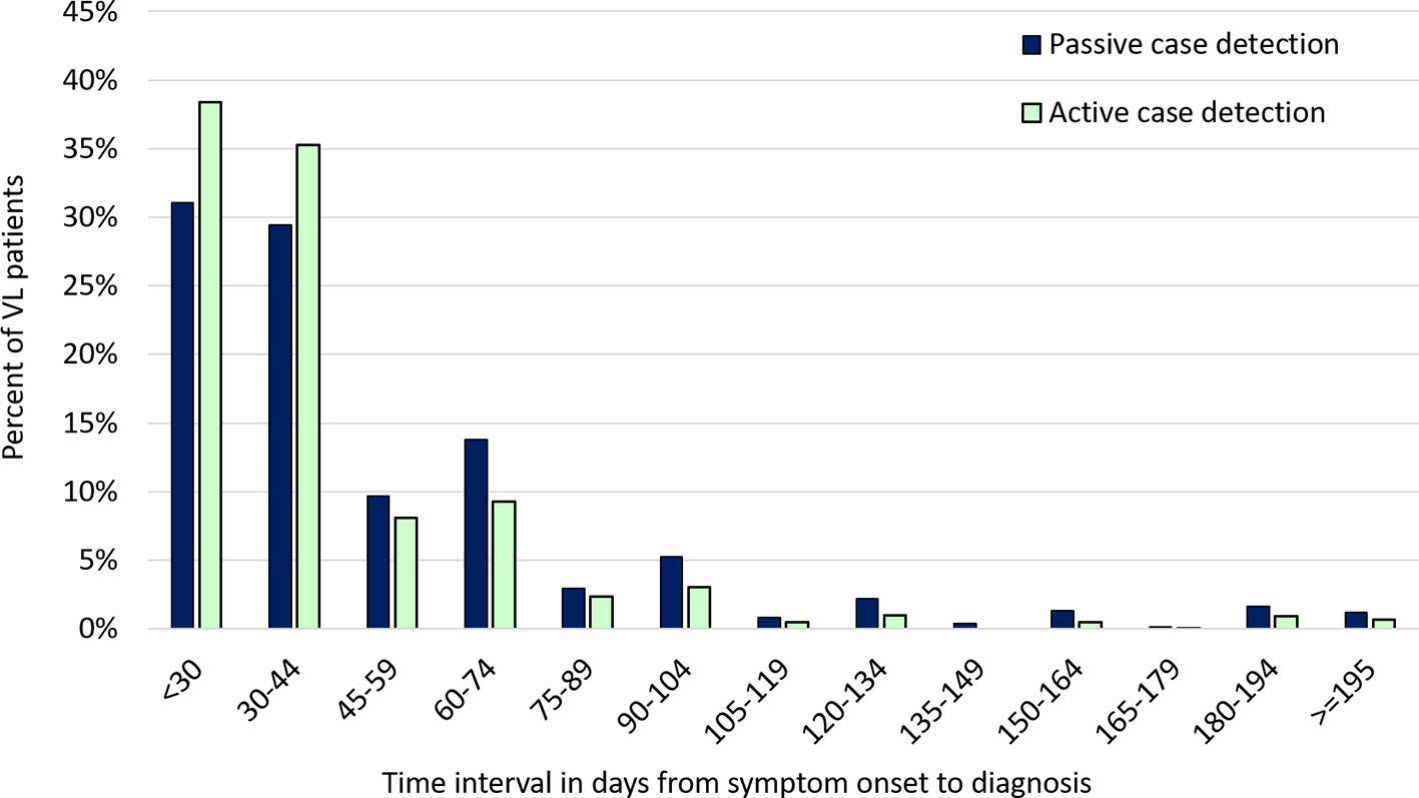
Distribution of symptom duration prior to diagnosis among visceral leishmaniasis patients identified by active versus passive case detection, January 1, 2018 - July 31, 2019. The figure shows the percentage of VL cases falling into each category of duration in days from fever onset to diagnosis. The distribution of VL patients identified by active case detection (ACD; in pale green) is shifted toward shorter duration categories compared to that for patients identified by passive case detection (PCD; in blue). The distribution has a very long right-hand tail, with a small percentage of patients having durations longer than 6 months; PCD predominates over ACD in the longer duration categories. The analysis includes 5030 patients reported to the Kala-azar Management Information System (KAMIS) during the time period.

The analysis dataset included 5030 VL patients. Males outnumbered females (57.9% vs 42.1%), 30.7% of patients were younger than 15 years and previous VL treatment was uncommon (8.7%) (**Table 1**). Close to 35% of patients came from marginalized castes and 37.7% reported living in a kuccha (unplastered mud brick) house. Based on case definitions, 3033 (60.3%) patients were detected passively and 1997 (39.7%) through ACD. House-to-house searches were conducted in 142 villages of 47 blocks in June-July of 2019; 15 VL patients were detected through these activities and the remaining 1982 VL cases were detected through the ongoing village-based key informant and snowballing method. The most frequent ACD referral sources were KBCs and ASHAs. The mean time from fever onset to diagnosis was 39.7 days (standard deviation 34.8 days) by ACD vs 49.3 days (SD 43.1 days) by PCD (p<0.0001). There was a strong tendency for patients to report fever duration as 20, 30, 45 or 60 days (median and mode 30 days for both ACD and PCD), but with a long right-hand tail (**Figure 6**). For that reason, subsequent analyses were performed using a categorical variable for duration of fever, and we relied on multinomial models to assess the effect of independent variables on diagnostic delays. Among all VL patients, 34.0%, 40.8%, 14.7% and 10.5% were diagnosed <30 days, 30-59 days, 60-89 and 90 days or more after fever onset. A higher percentage of patients detected by ACD fell into the earlier time intervals compared to those detected by PCD.

**Table 1:** Characteristics of 5030 visceral leishmaniasis (VL) patients included in the current analysis, and distribution of symptom duration in days prior to diagnosis, Bihar State, India, January 2018 – July 2019.

Patient characteristic	Total N	Duration of symptoms prior to VL diagnosis [n (Row %)]			
	(Column %)	<30 days	30–59 days	60–89 days	≥90 days
Total	5030 (100.0)	1710 (34.0)	2052 (40.8)	739 (14.7)	529 (10.5)
Age group					
<15 years	1544 (30.7)	609 (39.4)	617 (40.0)	203 (13.2)	115 (7.5)
15-35 years	1832 (36.4)	610 (33.3)	751 (41.0)	281 (15.3)	190 (10.4)
>35 years	1654 (32.9)	491 (29.7)	684 (41.4)	255 (15.4)	224 (13.5)
Sex					
Male	2912 (57.9)	995 (34.2)	1182 (40.6)	405 (13.9)	330 (11.3)
Female	2118 (42.1)	715 (33.8)	870 (41.1)	334 (15.8)	199 (9.4)
Previously treated for VL^1^					
Yes	435 (8.7)	145 (33.3)	180 (41.4)	61 (14.0)	49 (11.3)
No	4592 (91.4)	1565 (34.1)	1870 (40.7)	678 (14.8)	479 (10.4)
Caste type^2^					
Marginalized	1739 (34.7)	568 (32.7)	764 (43.9)	234 (13.5)	173 (10)
Non-marginalized	3279 (65.3)	1139 (34.7)	1284 (39.2)	503 (15.3)	353 (10.8)
House construction^3^					
Kuccha	1891 (37.7)	649 (34.3)	804 (42.5)	249 (13.2)	189 (10)
Pucca or Semi-pucca	3132 (62.4)	1059 (33.8)	1245 (39.8)	490 (15.7)	338 (10.8)
Mode of case detection					
Active	1997 (39.7)	767 (38.4)	865 (43.3)	232 (11.6)	133 (6.7)
Passive	3033 (60.3)	943 (31.1)	1187 (39.1)	507 (16.7)	396 (13.1)
ACD referral source^4^					
ASHA	676 (33.9)	250 (37.0)	306 (45.3)	79 (11.7)	41 (6.1)
KBC	847 (42.4)	319 (37.7)	375 (44.3)	99 (11.7)	54 (6.4)
Others	473 (23.7)	197 (41.7)	184 (38.9)	54 (11.4)	38 (8.0)
HIV infection status^5^					
Positive	193 (3.9)	52 (26.9)	63 (32.6)	35 (18.1)	43 (22.3)
Negative	4768 (96.1)	1628 (34.1)	1966 (41.2)	695 (14.6)	479 (10.1)

^1^ Data missing for 3 patients. ^2^ Data missing for 12 patients. ^3^ Data missing for 7 patients. ^4^Source of active case detection referral: KBC, kala-azar block coordinator; data missing for one patient. ^5^Data missing for 69 patients.

In univariable multinomial analyses, younger age and ACD were associated with significantly shorter duration and HIV infection with longer duration categories (**Table 2**). For other variables, associations were less consistent across time intervals. In the multivariable model, younger age and ACD remained associated with earlier and HIV with later diagnosis (**Table 3**). The advantage of ACD over PCD was more marked as the time interval increased: the adjusted odds ratios for illness durations of 30-59, 60-89 and ≥90 days, compared to the referent of <30 days, for ACD vs PCD were 0.88, 0.56 and 0.42 respectively. A total of 118 (2.4%) patients appear in the extreme right tail of the distribution in **Figure 5**, with delays of 180 days or longer. Extreme delays showed a significant association with older age, male sex, HIV infection and passive case detection in univariable analyses (**Supplemental Table S2**). In the adjusted model, HIV infection was a very strong risk factor for extreme delay, whereas younger age and ACD were associated with a significant protective effect.

**Table 2:** Univariable multinomial logistic regression models for factors associated with time from symptom onset to diagnosis among 5030 visceral leishmaniasis patients, Bihar State, India, January 2018 – July 2019.

Characteristic	Duration of symptoms prior to VL diagnosis					
	30-59 days		60-89 days		≥90 days	
	aOR (95%CI)	p-value	aOR (95%CI)	p-value	aOR (95%CI)	p-value
Age group						
<15 years	0.73 (0.62-0.85)	0.0001	0.64 (0.52-0.80)	<.0001	0.41 (0.32-0.53)	<0.0001
15 to 35 years	0.88 (0.76-1.04)	0.1245	0.89 (0.72-1.09)	0.2562	0.68 (0.54-0.86)	0.001
>35 years	Referent		Referent		Referent	
Sex						
Male	0.98 (0.86-1.11)	0.7176	0.87 (0.73-1.04)	0.1205	1.19 (0.98-1.46)	0.0865
Female	Referent		Referent		Referent	
Previously treated for VL^1^						
Yes	1.04 (0.83-1.31)	0.7437	0.97 (0.71-1.33)	0.8538	1.10 (0.79-1.55)	0.5677
No	Referent		Referent		Referent	
Caste type^2^						
Marginalized	1.19 (1.04-1.37)	0.0102	0.93 (0.78-1.12)	0.4614	0.98 (0.8-1.21)	0.8698
Non-marginalized	Referent		Referent		Referent	
House construction^3^						
Kuccha	1.05 (0.92-1.20)	0.4367	0.83 (0.69-0.99)	0.0427	0.91 (0.75-1.12)	0.3764
Pucca or Semi-pucca	Referent		Referent		Referent	
Mode of case detection						
Active	0.90 (0.79-1.02)	0.0962	0.56 (0.47-0.68)	<.0001	0.41 (0.33-0.51)	<0.0001
Passive	Referent		Referent		Referent	
ACD referral source						
ASHA	1.31 (1.01-1.70)	0.0426	1.15 (0.78-1.71)	0.4785	0.85 (0.53-1.37)	0.5067
KBC	1.26 (0.98-1.62)	0.0717	1.13 (0.78-1.65)	0.5177	0.88 (0.56-1.38)	0.5704
Others	Referent		Referent		Referent	
HIV infection status^4^						
Positive	1.00 (0.69-1.46)	0.99	1.58 (1.02-2.44)	0.041	2.81 (1.85-4.26)	<0.0001
Negative	Referent		Referent		Referent	

Intervals of 30-59, 60-89 and ≥90 days are compared to <30 days. ^1^Data missing for 3 patients.

^2^Data missing for 12 patients.

^3^Data missing for 7 patients.

^4^Data missing for 69 patients.

**Table 3:** Multivariable multinomial logistic regression model for factors associated with time from symptom onset to diagnosis among 4949 visceral leishmaniasis patients reported from January 2018 to July 2019 in Bihar State.

Characteristic	Duration of symptoms prior to VL diagnosis					
	30-59 days		60-89 days		≥90 days	
	aOR^1^ (95%CI)	p-value	aOR (95%CI)	p-value	aOR (95%CI)	p-value
Active Case Detection	0.88 (0.77-1.00)	0.06	0.56 (0.47-0.68)	<0.0001	0.42 (0.34-0.53)	<0.0001
Passive Case Detection	Referent		Referent		Referent	
Age group						
<15 years	0.69 (0.58-0.82)	<0.0001	0.64 (0.51-0.81)	0.0001	0.45 (0.34-0.58)	<0.0001
15 to 35 years	0.86 (0.73-1.01)	0.06	0.88 (0.71-1.09)	0.24	0.70 (0.56-0.89)	0.003
>35 years	Referent		Referent		Referent	
Male	0.93 (0.82-1.06)	0.29	0.81 (0.68-0.97)	0.03	1.02 (0.83-1.26)	0.82
Female	Referent		Referent		Referent	
Marginalized castes	1.28 (1.11-1.47)	0.0005	1.07 (0.88-1.29)	0.49	1.22 (0.98-1.51)	0.08
Non-marginalized castes	Referent		Referent		Referent	
HIV-positive	0.91 (0.62-1.32)	0.61	1.28 (0.82-2.01)	0.28	1.89 (1.23-2.90)	0.004
HIV-negative	Referent		Referent		Referent	

Eighty-nine patients with data missing for one or more variables are excluded from the model. Time intervals of 30-59, 60-89 and ≥90 days are compared to diagnosis <30 days after onset. ^1^Odds ratio adjusted for all listed variables.

## Discussion

The 2013 and 2015 assessments utilized a combination of methods applied at large scale to detect cases that were previously undetected or unreported, and yet failed to find the very large numbers of additional cases that research studies predicted (Singh et al., 2006; Singh et al., 2010). Two major differences may help to explain the very large difference in estimated under-reporting. Firstly, compared to the CARE assessments, the earlier studies covered much smaller populations, comprising 14 villages in one study and 17 villages in the other. Many of the apparently unreported cases may actually have been reported from adjacent blocks or districts. The CARE studies of 2013 and 2015 found such misclassification across geographies to be extensive, but after reconciling data from all geographies, the numbers of truly unreported cases turned out to be quite small in relation to the total number of reported cases. Secondly, both previous studies were conducted before the introduction of RDTs and miltefosine by the elimination program, whereas the CARE studies were conducted following comprehensive implementation of these commodities in the public sector. In the intervening period, there was likely a genuine massive shift of cases seeking care from private providers to seeking care from government facilities. The CARE studies did not attempt to cover the entire population of the affected districts in the two states, only those villages with evidence of a case. Thus, it is possible that we underestimated missing cases. There is not enough evidence available to estimate the magnitude of such underestimation, but later surveillance data suggest that it was relatively small.

The finding that at least 80% of all VL cases were already being captured by the system through passive case detection (PCD) methods (self-reporting of symptomatic cases to government facilities) meant that surveillance using any active case detection (ACD) would not be expected to add much to the total numbers. The main objective of any ACD methods deployed would therefore be to reduce the duration of symptoms before diagnosis, thus reducing the risk of transmission, a public health goal, and the risk of severe morbidity and mortality, important goals for individual patients. ACD methods were required that could be applied at program scale and able to effectively detect and screen symptomatic patients early.

In the 2018-2019 evaluation, active case detection identified nearly 40% of all VL cases and significantly decreased the time from fever onset to diagnosis. Notably, two-thirds of all patients had less than 60 days of illness, reflecting the major improvement in access to diagnosis and treatment that the VL elimination program has brought about over the last 10 years. In the early to mid-2000s, the median time to diagnosis was between 2 and 4 months in published studies from the Indian subcontinent, and access to rapid tests and highly effective treatment was limited (Ahluwalia et al., 2004; Mathur et al., 2005; Rijal et al., 2006). By 2010, after the introduction of miltefosine and rapid tests in the public health care system, the median duration of illness had already decreased to 40 days (Hasker et al., 2010; Pascual Martinez et al., 2012). In our data, the median time to diagnosis approached 30 days, and may not be susceptible to additional overall declines. Even more clinically important, ACD showed a more marked difference for longer time intervals, with nearly 60% lower odds of a patient being ill for 90 or more days, compared to those detected passively. The only other factor consistently and significantly associated with earlier diagnosis was younger age. This may reflect care-seeking based on parental concern, but possibly also a tendency toward more rapid progression of VL in children compared to adults (Urbane et al., 2019). Care seeking also may be more prompt for wage-earners than for older members of the household (Jayakumar et al., 2019). HIV-coinfected patients had substantially longer duration of VL prior to diagnosis. Indeed, HIV-infected patients had close to 4-fold increased odds of being ill for 6 months or more prior to diagnosis. This likely reflects a more prolonged search for care, as only a few medical centers in Bihar treat HIV-VL-coinfected patients (Burza et al., 2014). Diagnosis may also require invasive tests that are not widely available, as false-negative serology has been reported to be more frequent (Alvar et al., 2008). A similar phenomenon may have occurred for the small proportion of patients with a history of prior VL treatment, as guidelines recommend parasitological diagnosis and optimum re-treatment regimens may be less widely available (National Vector Borne Disease Control Programme, 2017).

One of the central challenges for the next phase of the VL elimination program is to ensure timely detection of individual cases and small outbreaks as incidence falls and the disease is no longer perceived as a major threat (Rijal et al., 2019). During the extensive VL assessments conducted in 2013-2015, two major observations led to the design of the ACD methodology evaluated here (Bindroo et al., 2021). First, more than half of all cases in a given year occurred in villages with cases in the previous year. Second, both geographic and social links can be used to increase the yield of cases. Based on these two facts, the resulting method focuses on villages with cases in the previous year and the use of local informants and snowballing based on known cases.

Unlike research studies that sought to evaluate snowballing techniques in a blinded fashion (Siddiqui et al., 2016), our program activities began with each known case. In contrast to the so-called index-case approach (Singh et al., 2011; Huda et al., 2012), we did not base the search on a fixed distance around the identified VL case, but sought other cases through the social networks of the known case, key informants active in health issues in the community, and through private practitioners, both formal and informal. In India, social links and caste connections have strong predictive value for where subsequent VL cases occur (Pascual Martinez et al., 2012; Bulstra et al., 2018), and ongoing links with the same key informants over years facilitate collaboration. Raising awareness in affected communities and improving the knowledge base of community health workers can also enhance both care-seeking and ACD efforts (Malaviya et al., 2013; Khatun et al., 2014).

Existing data, including ours, are insufficient to prove that ACD by itself has an impact on transmission and therefore future incidence of VL. Over the course of the elimination program, other interventions have been occurring, most importantly rapid effective diagnosis and treatment of cases at the primary health care level. In addition, there is a natural decrease in incidence over time at the end of an epidemic cycle. Rigorous assessment of the contributions of different interventions would require clinical trials of very large populations. However, the theoretical contribution of ACD to reducing time to diagnosis and thereby decreasing transmission is estimated to be substantial based on mathematical modeling (Medley et al., 2015).

Although we did not address PKDL data in this analysis, ACD for PKDL is an integral part of the ongoing field approach. PKDL patients are known to be infectious to sand flies and are widely considered to constitute an important inter-epidemic reservoir (Mondal et al., 2019). Verbal descriptions and pictures of PKDL skin lesions are used during case searches to orient local informants and enquire periodically about new cases. Attempts are underway to visit every VL patient listed in KAMIS once every six months. Once this effort has been underway for a sufficient period of time, these data could be used to determine optimal duration of follow-up and refine the ACD efforts for PKDL.

Thus, as discussed previously, the primary purpose of ACD going forward should be to reduce time to diagnosis. Early detection of suspects with fever of more than 15 days duration could be maximized by deploying ACD efforts in a given geography once every fortnight, if not continually. The methods described here are much more feasible to deploy at high frequency than house to house searches and were deployed to target all villages affected in the previous 12 months using a field team that is closely similar in size to the human resources available to the government health system. As incidence drops over time and the number of villages affected in the previous year declines, the same level of effort would cover villages affected over a wider past time-window, thus potentially increasing the yield of ACD even further. CARE teams work closely with government functionaries at all levels of the health department to train and facilitate use of these methods by the human resource available to the elimination program.

This evaluation was based on ongoing program data, rather than purpose-built research data collection, and as such, its strengths come with inevitable limitations. We observed clustering of duration data at 20, 30 and 60 days, indicating that patients were rounding to time intervals such as one or two months. Such rounding is likely unavoidable for a relatively chronic, insidious illness. We defined our analysis intervals to ensure that each category contained only one of these clustered values. Approximately 5% of cases reported in KAMIS lacked CDF data, either because the forms had not been completed or because identity code errors precluded linkage. The program has now implemented data collection using tablet computers with data checking software, integrated within KAMIS and this is applicable to any ACD method deployed; this will not only minimize such issues in the future but provide more timely access to the data. Our analysis suggests that the exclusions are unlikely to have introduced major biases that might affect the ACD vs PCD evaluation.

The term active case detection (ACD) has been used to describe a wide range of methodologies, ranging from fever camps run by mobile teams and geographically delimited searches around index cases to labor-intensive house-to-house surveys (Hirve et al., 2010; Das et al., 2014; Khatun et al., 2014; Banjara et al., 2015; Banjara et al., 2019; Huda et al., 2019; Singh et al., 2019). During the formulation of regional VL elimination guidelines, several head-to-head research studies were conducted to compare these different techniques (Singh et al., 2011; Huda et al., 2012; Hirve et al., 2017). Even in these early evaluations, the cost and yield for specific techniques varied widely. For example, in one WHO-supported study in the region, the cost per case detected ranged from $22 to $661 for the camp approach and from $50 to $540 for an incentive-based approach (Singh et al., 2011). Obviously, the yield for any ACD method has a strong inverse relationship to incidence and to the functioning of the health system: the more undiagnosed cases there are in the community, the easier it will be to find them (Mondal et al., 2009). Evaluations performed in the late 2000s when VL incidence in India topped 30,000 cases are not indicative of the yield and cost in the current setting of fewer than 4000 cases per year (Rijal et al., 2019). Indeed, due to the very low incidence in recent years, evaluations of ACD in Nepal and Bangladesh produced few cases, and some data suggest that treatment and reporting delays may be increasing (Banjara et al., 2019; Huda et al., 2019; Lim et al., 2019). These findings indicate that the next phase of the program may require an updated approach to surveillance and case detection.

The light-touch ACD strategies described help not only to reduce time to diagnosis, and thus risk of transmission, but also ensure a double check on the proportion of cases actually getting captured. These methods were evolved to be replicable with minimal resources. Currently, CARE is working in partnership with the government to prepare for the eventual transition to full government management in Bihar and Jharkhand. Both states have issued directives for program functionaries to be trained in these methods, and such trainings have been carried out in most districts. The elimination program may be able to sustain these methods for some years to come, possibly in combination with ACD for other diseases. Such a process can supplement passive case detection efforts that must go on, possibly perpetually, even after the elimination target is achieved.

## Data Availability Statement

The raw data supporting the conclusions of this article will be made available by the authors, without undue reservation.

## Ethics Statement

The 2013 and 2015 under-reporting assessment protocols were approved by the Institutional Committee for Ethics and Review of Health Management Research Office of Indian Institute of Health Management Research (IIHMR), Jaipur, India (www.iihmr.org). Informed consent (including signature or left thumb impression of the respondent) was obtained from each participant, after explaining the details of the study in a language that they could understand. The ethics committee of All India Institute of Medical Sciences-Patna approved the ACD effectiveness evaluation protocol for analysis of data from the VL surveillance system; no new data were collected under the research protocol.

## Author Contributions

The study was designed by PD, AD, KP, SS, JB, TM, CB, AOF. Data were curated, cleaned and analyzed by PD, AD, KP, JB, TM, AH, CB, LACC, SS and AOF. PD, CB, LACC and SS wrote the manuscript. All authors contributed to the article and approved the submitted version.

## Funding

This work was supported, in whole by the Bill & Melinda Gates Foundation [Grant ID# OPP1196454] http://www.gatesfoundation.org/. Under the grant conditions of the Foundation, a Creative Commons Attribution 4.0 Generic License has already been assigned to the Author Accepted Manuscript version that might arise from this submission. LACC was supported by the Bill and Melinda Gates Foundation through the Setting the Post-Elimination Agenda for Kala-azar in India consortium (Grant OPP1183986). The funders had no role in study design, data collection and analysis, decision to publish, or preparation of the manuscript.

## Conflict of Interest

The authors declare that the research was conducted in the absence of any commercial or financial relationships that could be construed as a potential conflict of interest.
