# Supplementary material for: Development and Evaluation of Active Case Detection Methods to Support Visceral Leishmaniasis Elimination in India
Source: Front Cell Infect Microbiol. 2021 Mar 24;11:648903. doi: 10.3389/fcimb.2021.648903 (PMC8024686; doi:10.3389/fcimb.2021.648903)
Supplement: Supplementary file 1 [file Table_1.docx]

Table S1. Comparison of demographics and other characteristics of visceral leishmaniasis patients with and without linked Case Details Form (CDF) data

|  | | With CDF data | Missing CDF data | |  |
| --- | --- | --- | --- | --- | --- |
|  | | N=5030 | N=251 | | P value^1^ |
| Age group (years) | |  |  | |  |
| <15 | | 1544 (95.8) | 68 (4.2) | |  |
| 15-35 | | 1832 (94.6) | 105 (5.4) | |  |
| >35 | | 1654 (95.5) | 78 (4.5) | | 0.93 |
| Sex | |  |  | |  |
| Male | | 2912 (94.8) | 161 (5.2) | |  |
| Female | | 2118 (95.9) | 90 (4.1) | | 0.06 |
| By reporting period | | |  | |  |
| Jan-June 2018 | | 1929 (93.0) | 145 (7.0) | |  |
| Jul-Dec 2018 | | 1453 (95.0) | 77 (5.0) | |  |
| Jan-June 2019 | | 1648 (98.3) | 29 (1.7) | | <0.0001 |
| Caste^2^ | |  |  | |  |
| Marginalized | | 1739 (96.0) | 72 (4.0) | |  |
| Other | | 3279 (94.8) | 179 (5.2) | | 0.06 |
| Previous VL treatment^3^ |  | | |  | |
| Yes | | 435 (89.0) | 54 (11.0) | |  |
| No | | 4592 (95.9) | 197 (4.1) | | <0.0001 |
| HIV infection status^4^ | |  |  | |  |
| Positive | | 193 (91.0) | 19 (9.0) | |  |
| Negative | | 4768 (95.5) | 223 (4.5) | | 0.002 |

^1^By Mantel-Haenszel Chi square test

^2^Data missing for 12 patients

^3^Data missing for 3 patients

^4^Data missing for 93 patients
