# Supplementary material for: Development and Evaluation of Active Case Detection Methods to Support Visceral Leishmaniasis Elimination in India
Source: Front Cell Infect Microbiol. 2021 Mar 24;11:648903. doi: 10.3389/fcimb.2021.648903 (PMC8024686; doi:10.3389/fcimb.2021.648903)
Supplement: Supplementary file 2 [file Table_2.docx]

Table S2. Comparison of patients and multivariable logistic regression model for factors associated with delays in diagnosis of 180 days or more, compared to delays of <180 days.

|  | >=180 days  N=118 | <180 days N=4912 | P value^1^ | Adjusted Odds ratio | 95% confidence intervals | P value |
| --- | --- | --- | --- | --- | --- | --- |
| Age group (years) |  |  |  |  |  |  |
| <15 | 17 (14.4%) | 1527 (31.1%) |  | 0.38 | 0.22, 0.68 | 0.001 |
| 15-35 | 41 (34.8%) | 1791 (36.5%) | <0.0001 | 0.69 | 0.46, 1.06 | 0.09 |
| >35 | 60 (50.9%) | 1594 (32.5%) |  | Referent |  |  |
| Sex |  |  |  |  |  |  |
| Male | 84 (71.2%) | 2828 (57.6%) | 0.003 | 1.45 | 0.96, 2.21 | 0.08 |
| Female | 34 (28.8%) | 2084 (42.4%) |  | Referent |  |  |
| Mode of detection |  |  |  |  |  |  |
| Active | 31 (26.3%) | 1966 (40.0%) | 0.003 | 0.60 | 0.39, 0.92 | 0.02 |
| Passive | 87 (73.7%) | 2946 (60.0%) |  | Referent |  |  |
| Caste |  |  |  |  |  |  |
| Marginalized | 37 (31.4%) | 1792 (34.7%) | 0.45 |  |  |  |
| Other | 81 (68.6%) | 3198 (65.3%) |  |  |  |  |
| Previously treated for VL |  |  |  |  |  |  |
| Yes | 15 (12.7%) | 420 (8.6%) | 0.13 |  |  |  |
| No | 193 (87.3%) | 4489 (91.4%) |  |  |  |  |
| HIV infection status^4^ |  |  |  |  |  |  |
| Positive | 21 (18.4%) | 172 (3.6%) | <0.0001 | 3.98 | 2.36, 6.69 | <0.0001 |
| Negative | 93 (81.6%) | 4675 (96.4%) |  | Referent |  |  |

^1^By Mantel-Haenszel Chi square or Fisher exact test.
